# Supplementary material for: Tradeoffs in hyphal traits determine mycelium architecture in saprobic fungi
Source: Sci Rep. 2019 Oct 2;9:14152. doi: 10.1038/s41598-019-50565-7 (PMC6775140; doi:10.1038/s41598-019-50565-7)
Supplement: Supplementary file 1 — Supplementary Information [file 41598_2019_50565_MOESM1_ESM.docx]

# Supplementary Information

Title: Tradeoffs in hyphal traits determine mycelium architecture in saprobic fungi

Anika Lehmann 1,2,*, Weishuang Zheng 3, Katharina Soutschek 1, Julien Roy 1,2, Andrey M. Yurkov 4, Matthias C. Rillig 1,2

1 Freie Universität Berlin, Institut für Biologie, Plant Ecology, Altensteinstr. 6, D-14195

Berlin, Germany;

2 Berlin-Brandenburg Institute of Advanced Biodiversity Research (BBIB), D-14195 Berlin,

Germany;

3 PKU-HKUST ShenZhen-Hong Kong Institution, Shenzhen 518057, China;

4 Leibniz Institute DSMZ - German Collection of Microorganisms and Cell Cultures, Inhoffenstraße 7B, D-38124 Braunschweig, Germany

* Corresponding author, Freie Universität Berlin, Institut für Biologie, Plant Ecology,

Altensteinstr. 6, D-14195 Berlin, Germany. Tel.: +49 30 83853145. Fax: 49 30 83853886.

E-mail address: lehmann.anika@googlemail.com

Keywords: saprobic fungi, traits, tradeoff, mycelium, architecture

## Diameter adjusted vs. not adjusted data


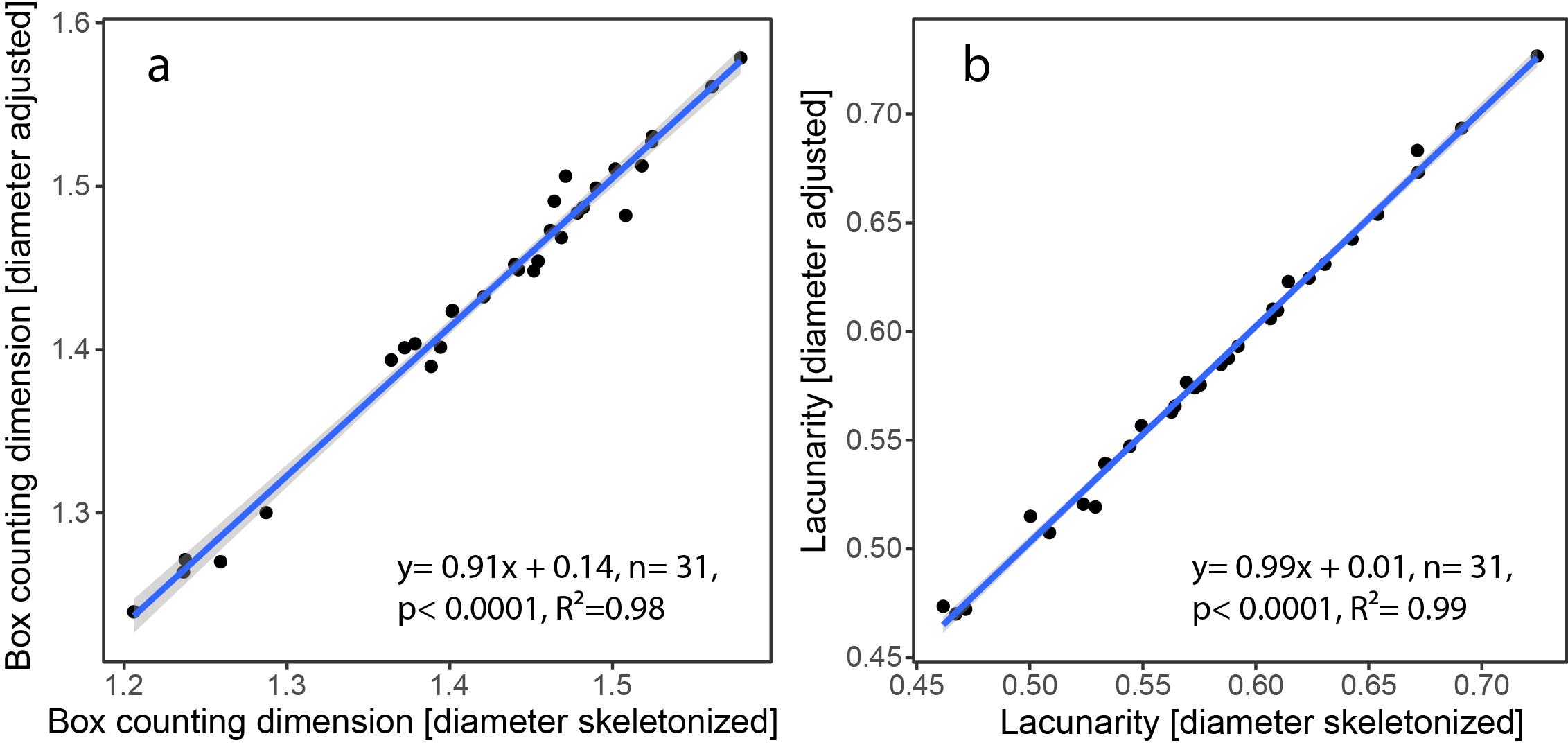


**Figure S1** Correlations of data derived from pictures with skeletonized (1 pixel wide for each strain) and adjusted diameter (adjusted by mean diameter) data. Data show a highly significant correlation.

## Information on fungal strains

**Table S1** Information about phylum, order, taxon name and Deutsche Sammlung von Mikroorganismen und Zellkulturen (German Collection of Microorganisms and Cell Cultures GmbH, DSMZ) accession numbers of the 31 fungal strains used in this study.

| **strain ID** | **DSMZ accession number** | **Phylum** | **Order** | **Taxon identification *** |
| --- | --- | --- | --- | --- |
| RLCS10 | DSM100286 | Ascomycota | Pleosporales | Alternaria alternata |
| RLCS21 | DSM100327 | Ascomycota | Pleosporales | Pyrenochaetopsis leptospora |
| RLCS22 | DSM100401 | Ascomycota | Pleosporales | Paraphoma chrysanthemicola |
| RLCS12 | DSM100405 | Ascomycota | Pleosporales | Didymellaceae strain 1 |
| RLCS14 | DSM100404 | Ascomycota | Pleosporales | Didymellaceae strain 2 |
| RLCS30 | DSM100291 | Ascomycota | Chaetothyriales | Exophiala equina |
| RLCS31 | DSM100328 | Ascomycota | Chaetothyriales | Cyphellophora sp. |
| RLCS28 | DSM100323 | Ascomycota | Helotiales | Tricladium sp. |
| RLCS26 | DSM100330 | Ascomycota | Helotiales | Tetracladium marchalianum |
| RLCS25 | DSM100292 | Ascomycota | Hypocreales | Hydropisphaera sp. |
| RLCS20 | DSM100329 | Ascomycota | Hypocreales | Purpureocillium lilacinum |
| RLCS24 | DSM100410 | Ascomycota | Hypocreales | Metarhizium marquandii |
| RLCS23 | DSM101519 | Ascomycota | Hypocreales | Stachybotryaceae strain 1 |
| RLCS05 | DSM100403 | Ascomycota | Hypocreales | Fusarium sp. |
| PLCS32 | DSM100409 | Ascomycota | Hypocreales | Fusarium oxysporum |
| RLCS08 | DSM100325 | Ascomycota | Hypocreales | Gibberella tricincta |
| RLCS18 | DSM100287 | Ascomycota | Hypocreales | Gibberella sp. |
| RLCS13 | DSM100290 | Ascomycota | Hypocreales | Fusarium solani |
| RLCS27 | DSM100326 | Ascomycota | Sordariales | Thielavia inaequalis |
| RLCS06 | DSM100400 | Ascomycota | Sordariales | Chaetomium angustispirale |
| RLCS07 | DSM100284 | Ascomycota | Xylariales | Amphisphaeriaceae strain 1 |
| RLCS29 | DSM100288 | Basidiomycota | Agaricales | Macrolepiota excoriata |
| RLCS17 | DSM100324 | Basidiomycota | Agaricales | Clitopilus sp. |
| RLCS16 | DSM100408 | Basidiomycota | Agaricales | Pleurotus pulmonarius |
| RLCS09 | DSM100406 | Basidiomycota | Polyporales | Trametes versicolor |
| RLCS03 | DSM100285 | Mucoromycota | Mortierellales | Mortierella alpina strain 1 |
| RLCS11 | DSM100289 | Mucoromycota | Mortierellales | Mortierella alpina strain 2 |
| RLCS15 | DSM100402 | Mucoromycota | Mortierellales | Mortierella elongata strain 1 |
| RLCS02 | DSM100407 | Mucoromycota | Mortierellales | Mortierella elongata strain 2 |
| RLCS04 | DSM100322 | Mucoromycota | Mortierellales | Mortierella exigua |
| RLCS01 | DSM100293 | Mucoromycota | Mucorales | Mucor fragilis |
| RLCS19 | DSM100331 | Mucoromycota | Umbelopsidales | Umbelopsis isabellina |

*best resolved tree annotation passing 80% threshold of bootstrap approach

Trait value differences among phyla

To evaluate differences in trait values for the three phyla, we run analyses of variance (Table S2) by using the aov() function with subsequent pairwise comparison (TukeyHSD test) for significant outcomes.

**Table S2** Analysis of Variance outcome for the different traits and the explanatory variable “phylum”.

| **traits** | **df** | **F value** | **p value** |
| --- | --- | --- | --- |
| Db | 2, 28 | 12.3 | 0.0001 |
| Dbcv | 2, 28 | 11.05 | 0.0003 |
| L | 2, 28 | 3.63 | 0.04 |
| Lcv | 2, 28 | 5.17 | 0.01 |
| BA | 2, 28 | 10.17 | 0.0005 |
| BAcv | 2, 28 | 4.407 | 0.02 |
| D | 2, 28 | 0.673 | 0.52 |
| Dcv | 2, 28 | 0.673 | 0.52 |
| IL | 2, 28 | 10.86 | 0.0003 |
| ILcv | 2, 28 | 2.352 | 0.11 |


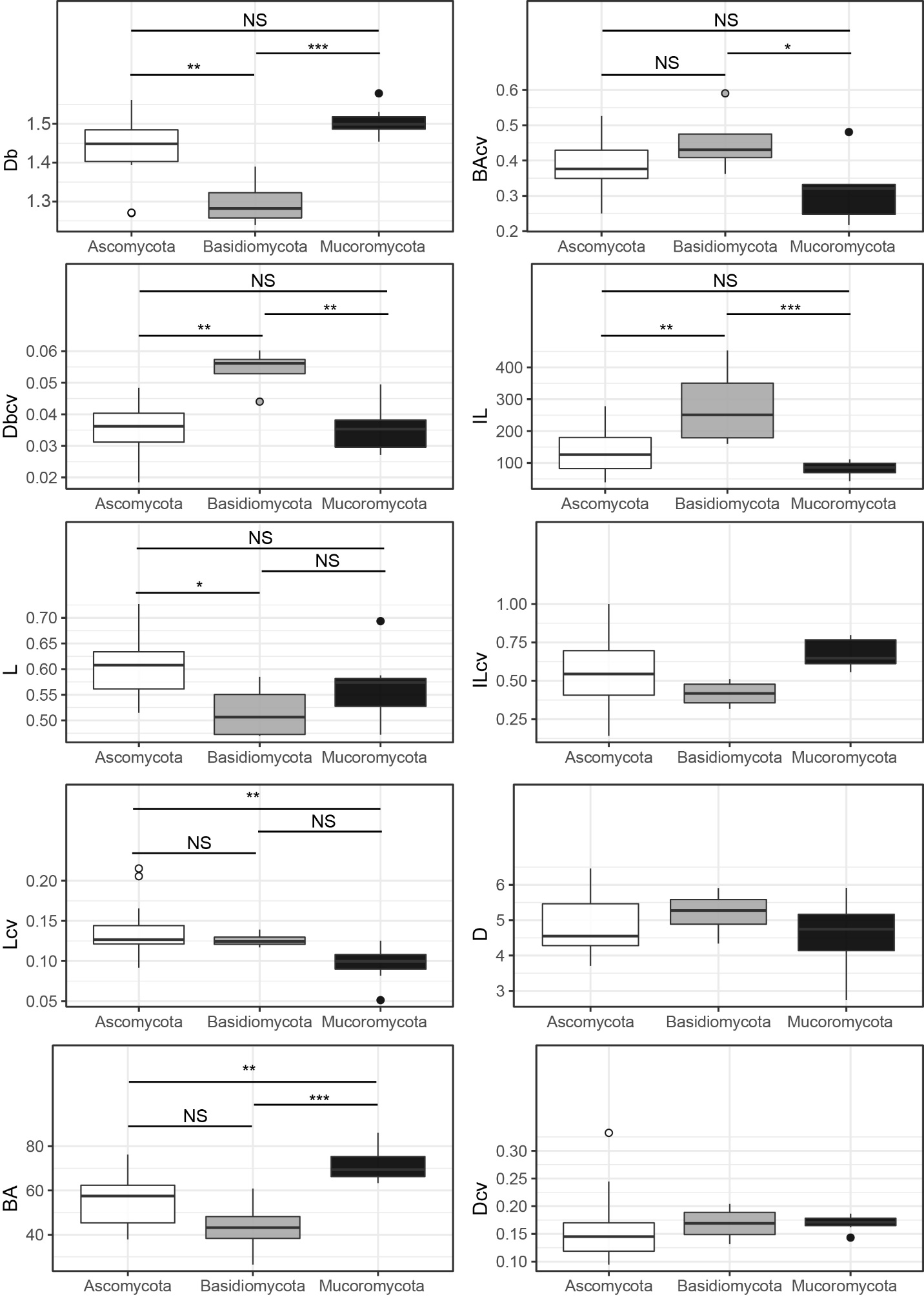


**Figure S2** Pairwise analysis outcomes of trait differences among phyla. Asterisks denote significance level: *** < 0.001, ** <0.01, * < 0.05. Outcomes of associated analysis of variance can be found in Table S2.

## PCA

**Table S3** Test for PC axis significance (class: krandtest lightkrandtest, Monte-Carlo tests, 999 permutations).

| PCA | Obs | Std.Obs | p-value |
| --- | --- | --- | --- |
| Axis 1 | 0.76977 | 5.82337 | 0.001 |
| Axis 2 | 0.62794 | 1.17790 | 0.123 |
| Axis 3 | 0.63817 | 1.41345 | 0.087 |
| Axis 4 | 0.72395 | 3.05316 | 0.004 |
| Axis 5 | 0.77264 | 3.25753 | 0.002 |
| Axis 6 | 0.59664 | 0.62184 | 0.259 |
| Axis 7 | 0.61490 | 0.03628 | 0.468 |
| Axis 8 | 0.67486 | 0.75227 | 0.226 |
| Axis 9 | 0.70232 | 0.45138 | 0.290 |
| Axis 10 | 0.84284 | 3.07740 | 0.002 |
| Axis 11 | 1.00000 | 6.44368 | 0.001 |

## Phylogenetic signal in architectural traits

**Table S4** Phylogenetic signal estimated by Moran’s I using R package “phylosignal”.

| Trait | I | p-value |
| --- | --- | --- |
| **Db** | **0.094909** | **0.038** |
| Db_CV_ | -0.02724 | 0.414 |
| L | -0.05457 | 0.653 |
| **L_CV_** | **0.102708** | **0.028** |
| BA | 0.036896 | 0.151 |
| BA_CV_ | -0.01455 | 0.367 |
| D | -0.11682 | 0.909 |
| D_CV_ | -0.02723 | 0.437 |
| IL | 0.001451 | 0.213 |
| IL_CV_ | -0.0045 | 0.305 |

## Relationship between phylum and PCA1


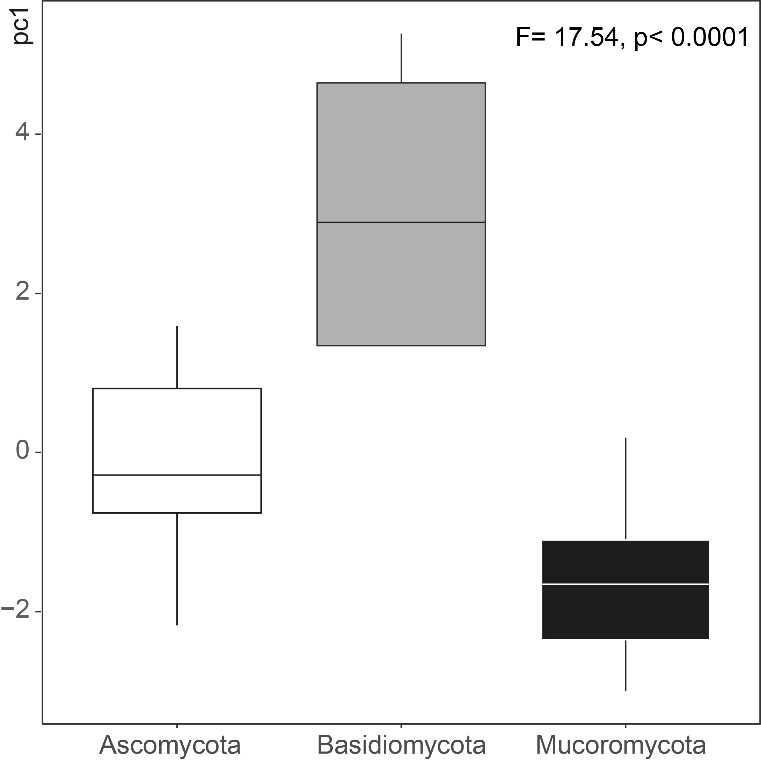


**Figure S3** Separation of phyla alongside PC axis 1, as the significant representative of the ten-dimensional trait space. Difference between phyla for PC1 were tested by analysis of variance. TukeyHSD test revealed that all pairwise comparisons were significantly different: B-A: p<000.1; M-A: p=0.02; M-B: p<0.0001.

## Pairwise relationships


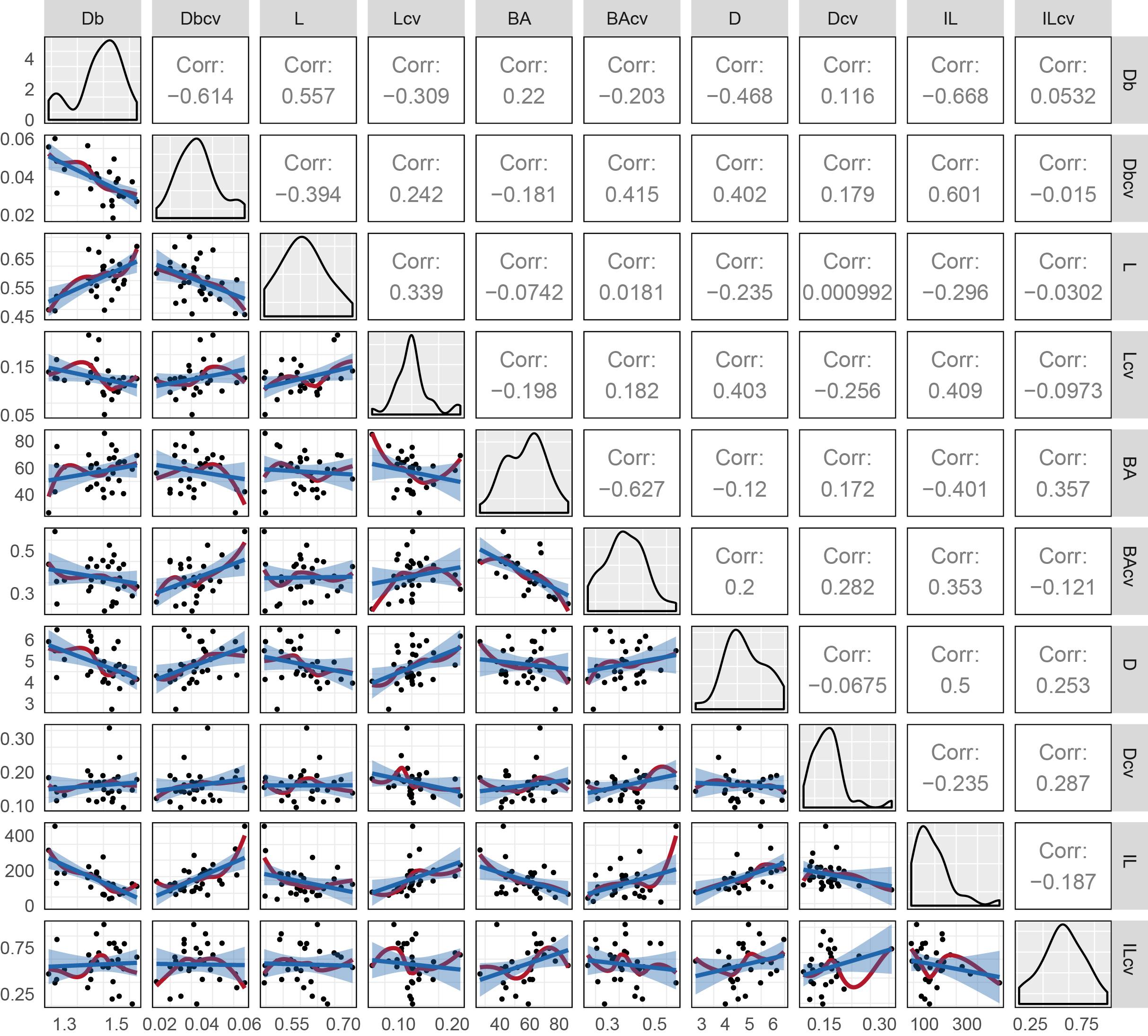


**Figure S4** Multiple pairwise relationships of the ten architectural traits. In the upper triangle, the correlation coefficients (Pearson’s rho) and, in the lower triangle, scatterplots of trait pairs are depicted. The red line represents loess and blue line linear regression line with corresponding confidence interval (blue shade). On the diagonal, histograms of the frequency distribution of the trait values are shown. Analyses were conducted on trait mean data (n= 31).

**
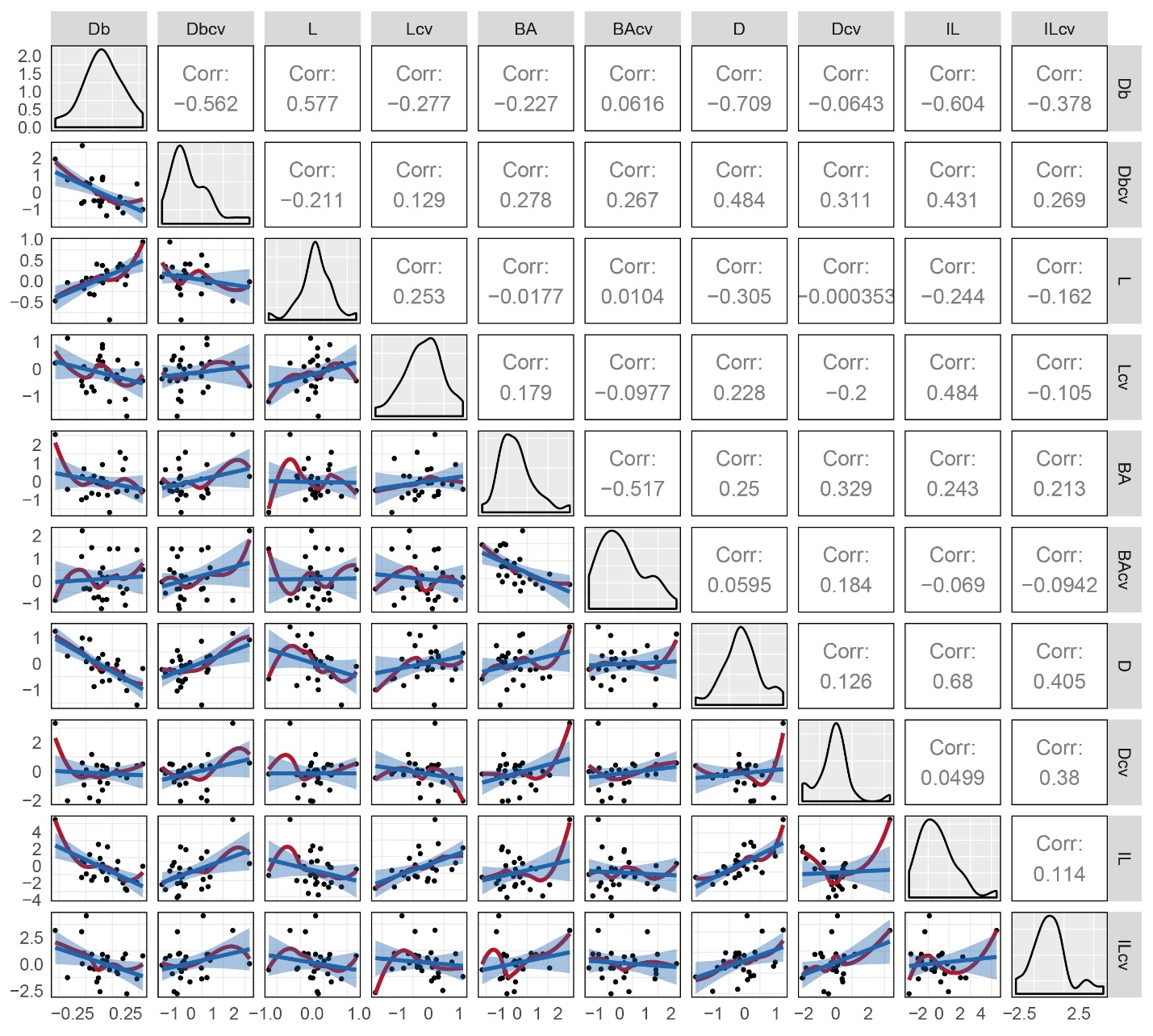
**

**Figure S5** Multiple pairwise relationships of phylogenetic corrected data (log-transformed) of the ten architectural traits. In the upper triangle, the correlation coefficients (Pearson’s rho) and, in the lower triangle, scatterplots of trait pairs are depicted. The red line represents loess and blue line linear regression line with corresponding confidence interval (blue shade). On the diagonal, histograms of the frequency distribution of the trait values are shown. Analyses were conducted on trait mean data (n= 28).

##

|  |  |  |  |
| --- | --- | --- | --- |

## Quantile and linear regression

**Table S5** Regression statistics of linear and quantile regression for all trait combinations with a |Pearson’s rho| > 0.30 and a significance level α< 0.05. The first eight trait pairs represent analysis outcomes for regressions depicted in Fig. 2c to 2j.

|  |  | Linear regression | | | Quantile regression | | |
| --- | --- | --- | --- | --- | --- | --- | --- |
| trait1 | trait2 | equation | R²adj | p | quantile | equation | p |
| Db | Db_CV_ | y= -0.61x - 0 | 0.36 | 0.0002 | 0.05 | y= -0.45x - 1.38 | 0.22 |
|  |  |  |  |  | 0.25 | y= -0.68x - 0.37 | 0.01 |
|  |  |  |  |  | 0.75 | y= -0.56x - 0.57 | 0.05 |
|  |  |  |  |  | 0.95 | y= -0.43x + 1.13 | 0.18 |
|  |  |  |  |  |  |  |  |
| Db | L | y= 0.56x - 0 | 0.29 | 0.001 | 0.05 | y= 0.56x - 1.35 | 0.002 |
|  |  |  |  |  | 0.25 | y= 0.81x - 0.56 | 0.02 |
|  |  |  |  |  | 0.75 | y= 0.29x 0.68 | 0.3 |
|  |  |  |  |  | 0.95 | y= 0.30x + 1.09 | 0.09 |
|  |  |  |  |  |  |  |  |
| Db | D | y= -0.47x - 0 | 0.19 | 0.008 | 0.05 | y= -0.18x - 2.00 | 0.67 |
|  |  |  |  |  | 0.25 | y= -0.65x - 0.65 | 0.05 |
|  |  |  |  |  | 0.75 | y= -0.63x + 0.64 | 0.02 |
|  |  |  |  |  | 0.95 | y= -0.40x + 1.28 | 0.04 |
|  |  |  |  |  |  |  |  |
| BA | BA_CV_ | y= -0.63x + 0 | 0.37 | 0.0002 | 0.05 | y= -1.02x - 1.44 | 0.03 |
|  |  |  |  |  | 0.25 | y= -1.02x - 0.31 | 0.001 |
|  |  |  |  |  | 0.75 | y= -0.64x + 0.47 | 0.04 |
|  |  |  |  |  | 0.95 | y= -0.39x + 1.40 | 0.05 |
|  |  |  |  |  |  |  |  |
| IL | Db | y= -0.67x - 0 | 0.43 | 0.0001 | 0.05 | y= -0.40x - 0.54 | 0.04 |
|  |  |  |  |  | 0.25 | y= -0.37x - 0.54 | 0.08 |
|  |  |  |  |  | 0.75 | y= -0.78x + 0.28 | 0.01 |
|  |  |  |  |  | 0.95 | y= -1.10x + 1.42 | 0.01 |
|  |  |  |  |  |  |  |  |
| IL | DB_CV_ | y= 0.60x + 0 | 0.34 | 0.0004 | 0.05 | y= 0.25x - 0.84 | 0.28 |
|  |  |  |  |  | 0.25 | y= 0.40x - 0.59 | 0.05 |
|  |  |  |  |  | 0.75 | y= 0.68x + 0.41 | 0.04 |
|  |  |  |  |  | 0.95 | y= 0.87x + 1.53 | 0.02 |
|  |  |  |  |  |  |  |  |
| IL | D | y= 0.50x - 0 | 0.22 | 0.004 | 0.05 | y= 0.15x - 0.89 | 0.51 |
|  |  |  |  |  | 0.25 | y= 0.29x - 0.59 | 0.05 |
|  |  |  |  |  | 0.75 | y= 0.43x + 0.24 | 0.1 |
|  |  |  |  |  | 0.95 | y= 1.33x + 2.11 | 0.04 |
|  |  |  |  |  |  |  |  |
| Dbcv | BA_CV_ | y= 0.42x - 0 | 0.14 | 0.02 | 0.05 | y= 0.30x - 1.61 | 0.27 |
|  |  |  |  |  | 0.25 | y= 0.23x - 0.64 | 0.47 |
|  |  |  |  |  | 0.75 | y= 0.49x + 0.62 | 0.04 |
|  |  |  |  |  | 0.95 | y= 0.90x + 1.42 | 0.0002 |
|  |  |  |  |  |  |  |  |
| Db | L_CV_ | y= -0.31x - 0 | 0.06 | 0.09 | 0.05 | y= -0.89x -1.86 | 0.12 |
|  |  |  |  |  | 0.25 | y= -0.22x - 0.31 | 0.55 |
|  |  |  |  |  | 0.75 | y= -0.25x + 0.60 | 0.16 |
|  |  |  |  |  | 0.95 | y= -0.59x + 1.42 | 0.31 |
|  |  |  |  |  |  |  |  |
| Db_CV_ | L | y= 0.30x - 0 | 0.13 | 0.03 | 0.05 | y= -0.16x - 1.24 | 0.62 |
|  |  |  |  |  | 0.25 | y= -0.25x - 0.68 | 0.4 |
|  |  |  |  |  | 0.75 | y= -0.44x + 0.58 | 0.14 |
|  |  |  |  |  | 0.95 | y= -0.48x + 1.51 | 0.08 |
|  |  |  |  |  |  |  |  |
| Db_CV_ | D | y= 0.40x - 0 | 0.13 | 0.03 | 0.05 | y= 0.31x -1.23 | 0.25 |
|  |  |  |  |  | 0.25 | y= 0.20x - 0.71 | 0.41 |
|  |  |  |  |  | 0.75 | y= 0.61x + 0.49 | 0.07 |
|  |  |  |  |  | 0.95 | y= 0.29x + 2.11 | 0.48 |
|  |  |  |  |  |  |  |  |
| L | L_CV_ | y= 0.34x - 0 | 0.08 | 0.06 | 0.05 | y= 0.01x - 1.66 | 0.98 |
|  |  |  |  |  | 0.25 | y= 0.35x - 0.67 | 0.24 |
|  |  |  |  |  | 0.75 | y= 0.25x + 0.84 | 0.42 |
|  |  |  |  |  | 0.95 | y= 1.01x + 1.71 | 0.03 |
|  |  |  |  |  |  |  |  |
| L_CV_ | D | y= 0.40x + 0 | 0.13 | 0.03 | 0.05 | y= 0.71x -1.74 | 0.05 |
|  |  |  |  |  | 0.25 | y= 0.32x - 0.59 | 0.13 |
|  |  |  |  |  | 0.75 | y= 0.36x + 0.28 | 0.25 |
|  |  |  |  |  | 0.95 | y= 0.63x + 1.66 | 0.31 |
|  |  |  |  |  |  |  |  |
| L_CV_ | IL | y= 0.41x + 0 | 0.14 | 0.02 | 0.05 | y= 0.30x -1.03 | 0.28 |
|  |  |  |  |  | 0.25 | y= 0.31x -0.63 | 0.16 |
|  |  |  |  |  | 0.75 | y= 0.71x + 0.52 | 0.18 |
|  |  |  |  |  | 0.95 | y= 1.50x + 1.43 | 0.06 |
|  |  |  |  |  |  |  |  |
| BA | IL | y= -0.40x + 0 | 0.13 | 0.03 | 0.05 | y= -0.8x - 0.99 | 0.45 |
|  |  |  |  |  | 0.25 | y= -0.12x - 0.66 | 0.57 |
|  |  |  |  |  | 0.75 | y= -0.66x + 0.46 | 0.09 |
|  |  |  |  |  | 0.95 | y= -1.21x + 2.20 | 0.01 |
|  |  |  |  |  |  |  |  |
| BA_CV_ | IL | y= 0.35x + 0 | 0.09 | 0.05 | 0.05 | y= -0.01x - 1.13 | 0.89 |
|  |  |  |  |  | 0.25 | y= 0.09x -0.66 | 0.65 |
|  |  |  |  |  | 0.75 | y= 0.41x + 0.34 | 0.37 |
|  |  |  |  |  | 0.95 | y= 0.77x + 1.63 | 0.06 |
|  |  |  |  |  |  |  |  |
| D | Db_CV_ | y= 0.60x + 0 | 0.34 | 0.0004 | 0.05 | y= 0.28x - 1.13 | 0.31 |
|  |  |  |  |  | 0.25 | y= 0.34x - 0.49 | 0.22 |
|  |  |  |  |  | 0.75 | y= 0.52x - 0.49 | 0.08 |
|  |  |  |  |  | 0.95 | y= -0.6x + 1.83 | 0.88 |
|  |  |  |  |  |  |  |  |
| IL_CV_ | BA | y= 0.36x - 0 | 0.1 | 0.04 | 0.05 | y= 0.62x - 1.37 | 0.26 |
|  |  |  |  |  | 0.25 | y= 0.38x - 0.85 | 0.21 |
|  |  |  |  |  | 0.75 | y= 0.55x + 0.55 | 0.06 |
|  |  |  |  |  | 0.95 | y= -0.28x + 1.70 | 0.61 |
